# 222 nm far-UVC efficiently introduces nerve damage in *Caenorhabditis elegans*

**DOI:** 10.1371/journal.pone.0281162

**Published:** 2023-01-31

**Authors:** Kaoru Okamoto Yoshiyama, Norihiko L. Okamoto, Jun Hidema, Atsushi Higashitani

**Affiliations:** 1 Graduate School of Life Sciences, Tohoku University, Sendai, Japan; 2 Institute for Materials Research, Tohoku University, Sendai, Japan; 3 Division for the Establishment of Frontier Sciences of the Organization for Advanced Studies, Tohoku University, Sendai, Japan; Lancaster University, UNITED KINGDOM

## Abstract

Far-ultraviolet radiation C light (far-UVC; 222 nm wavelength) has received attention as a safer light for killing pathogenic bacteria and viruses, as no or little DNA damage is observed after irradiation in mammalian skin models. Far-UVC does not penetrate deeply into tissues; therefore, it cannot reach the underlying critical basal cells. However, it was unclear whether far-UVC (222-UVC) irradiation could cause more biological damage at shallower depths than the 254 nm UVC irradiation (254-UVC), which penetrates more deeply. This study investigated the biological effects of 222- and 254-UVC on the small and transparent model organism *Caenorhabditis elegans*. At the same energy level of irradiation, 222-UVC introduced slightly less cyclobutane pyrimidine dimer damage to naked DNA in solution than 254-UVC. The survival of eggs laid during 0–4 h after irradiation showed a marked decrease with 254-UVC but not 222-UVC. In addition, defect of chromosomal condensation was observed in a full-grown oocyte by 254-UVC irradiation. In contrast, 222-UVC had a significant effect on the loss of motility of *C*. *elegans*. The sensory nervous system, which includes dopamine CEP and PVD neurons on the body surface, was severely damaged by 222-UVC, but not by the same dose of 254-UVC. Interestingly, increasing 254-UVC irradiation by about 10-fold causes similar damage to CEP neurons. These results suggest that 222-UVC is less penetrating, so energy transfer occurs more effectively in tissues near the surface, causing more severe damage than 254-UVC.

## Introduction

It is known that 254 nm ultraviolet radiation C light (254-UVC) induces cyclobutane pyrimidine dimers (CPDs) and 6–4 photoproducts (6-4PPs) at the DNA/RNA dipyrimidine sites, and these DNA/RNA lesions distort the DNA double-helix structure, lose sequence information, and severely prevent vital processes, such as replication and transcription [1, 2]. Thus, 254-UVC can kill microorganisms and cause great damage to human skin. Recently, not only germicidal 254-UVC but also far-UV radiation C light (far-UVC; 200–230 nm) efficiently kills pathogenic bacteria and viruses, such as methicillin-resistant *Staphylococcus aureus* and severe acute respiratory syndrome coronavirus-2 (SARS-CoV-2) [3, 4].

Far-UVC has been receiving a lot of attention recently. Far-UVC is harmless to the human body because these wavelengths have a very limited penetration depth and are less than a few micrometers. Thus, it cannot reach living human cells in the skin or eyes, as it is absorbed in the outer dead-cell skin or ocular tear layer [5–8]. The effects of far-UVC are based on the fact that UV light at a wavelength of around 200 nm is very strongly absorbed by proteins (particularly through the peptide bond) and other biomolecules [9–11]; therefore, its ability to penetrate the biological material is very limited. Sixty percent of the incident radiation at 193 nm UV light is absorbed within 1 μm of the cell surface by calculating the coefficient of molar absorbance for the peptide bond and aromatic amino acid at 193 nm UV [11]. Thus, far-UVC can penetrate bacteria and viruses that are typically <1 μm in size [5, 12], but it cannot penetrate the human stratum corneum (the outer dead-cell skin layer; thickness 5–20 μm) nor the ocular cornea (thickness ~500 μm) nor the cytoplasm of individual human cells [7, 13, 14]. In contrast, 254-UVC can be a health hazard to the skin and eyes because 254-UVC penetrates down to the basal cell layer, the bottom-most layer of the skin, and damages DNA [7]; therefore, it causes human skin cancer and cataracts [15–18]. The cross-sectional images of the skin samples from UVC-irradiated mice showed that 254-UVC exposure induced CPD and 6-4PPs, whereas skin exposed to 222-UVC did not induce either CPD or 6-4PPs [5]. Therefore, 222 nm UVC (222-UVC) seems an important tool to prevent infection without inducing mammalian skin damage. However, knowledge of the biological effects of 222-UVC is still limited. To further develop 222-UVC, it is necessary to know the biological effects of far-UVC on many organisms. Guard cells on the plant leaf surface are more affected by far-UVC than 254-UVC [19]. It also needs to be proven whether far-UVC can induce the photoproducts of DNA/RNA like 254-UVC.

*Caenorhabditis elegans* (*C*. *elegans*) is a multicellular genetic model organism because it shares many similarities with human tissues, but it is anatomically simpler than humans. The *C*. *elegans* (~1 mm long in adulthood) is much smaller than mammalian tissues, and its body is transparent; therefore, light easily passes through its body. Due to its transparency, the inside of tissues and cells can be observed in detail with a Nomarski microscope or a fluorescence microscope using Green Fluorescent Protein (GFP) fusion proteins. Using these advantages of *C*. *elegans*, we evaluated the biological effects of far-UVC on it. In particular, this study focused on the effects of far-UVC on sensory neurons near the body surface and oocyte and early embryo in the uterus of gravid adult hermaphrodites compared to 254-UVC. In addition, the efficiency of induction of UV-induced CPDs by 222- and 254-UVC on naked DNA was compared.

## Materials and methods

### Nematode strains and genetics

The *C*. *elegans* were cultured at 20°C on NGM plates seeded with *Escherichia coli* (OP50 strain). The N2 Bristol strain was used as the wild-type (WT) reference strain. The NC1686 *wdIs51* [*F49H12*.*4*::GFP + *unc-119* (+)] [20] and TG2435 *vtIs1* [*dat-1p*::GFP + *rol6* (*su1006*)] [21] were used in this study. All studies in this work used *C*. *elegans* hermaphrodites.

### Light sources

The UVC radiation used in this study was provided by a germicidal lamp (254-UVC; Toshiba GL20; Toshiba Electric Ltd., Tokyo, Japan) at a distance of 20 cm (6.8 J/m^2^/s) (100 J/m^2^: 15 s, 200 J/m^2^: 29 s). The far-UVC source used in this study was a 15 W 222 nm KrCl excimer lamp (222-UVC) made by ORC Manufacturing Co., Ltd. (Tokyo, Japan), and 222-UVC was provided at a distance of 12.5 cm (1.7J/m^2^/s) (100 J/m^2^: 60 s, 200 J/m^2^: 120 s). The UVC irradiation was performed in an open dark space set at 23°C. The spectra and energy fluence rates were measured with a spectroradiometer (USR-45DA; Ushio, Inc., Tokyo, Japan). All experiments were conducted using synchronized cultured nematodes. Synchronized adults were placed on the surface of an agar medium coated with *E*. *coli*, and UVC irradiation was performed directly on the nematode surface from the top after removing the lid of the plastic petri dish.

### T4 endonuclease assay

To know the amount of CPDs, this study used T4 endonuclease V, a pyrimidine dimer glycosylase (New England Biolabs, Ipswich, MA, USA) as described before with some modifications [22]. The UVC-irradiated and unirradiated lambda DNA were digested with T4 endonuclease in an attached buffer and incubated for 1 h at 37°C. The DNA was denatured by the addition of an alkaline stop mixture [0.5 M NaOH, 25% (v/v) glycerol, and 0.25% (w/v) bromocresol green] and incubation for 30 min 37°C. The DNA was separated by alkaline agarose gel [0.7% (w/v) Seakem GTG agarose (Lonza, Basel, Switzerland), 1 mM EDTA, 50 mM NaCl] electrophoresis (50V, 2h, 15°C) in buffer (30 mM NaOH and 2 mM EDTA) at15°C. The molecular length marker was lambda DNA with *Hin*d III digested lambda DNA (48.5, 23.1, 9.4, 6.6, 4.3, and 2.3 kb). The gel was neutralized for 30 min twice (0.1 M Tris-HCl, pH 7.5) and stained with SYBR Green II (Lonza) for 2 h. The experiment was dependently performed thrice.

### Hatching rate

Five synchronized gravid adult hermaphrodite worms were transferred to a new OP50/NGM with *E*. *coli* plates immediately before UVC irradiation and exposed to 254- or 222-UVC. The hatchability of eggs laid 4 hours after irradiation was scored (the adults were removed after 4 hours). The number of eggs that hatched overnight was then scored and calculated as hatchability [23]. The hatching rate was calculated as following formula (hatching rate = the number of hatching eggs / the number of laid eggs x 100) (*n* = 60–200).

### DAPI staining

To visualize chromosome, the animals were fixed in Carnoy’s solution (ethanol 3 ml, acetic acid 1.5 ml chloroform 0.5 ml) for several minutes and washed by M9 solution twice and stained with VECTORSHIELD Mounting Medium (funakoshi, Tokyo, JAPAN) [23]. Four hours after UVC irradiation, oocytes and fertilized eggs in uterus of adult worms were observed with a confocal laser scanning microscopy (FV10i, OLYMPUS). Oocytes and eggs were imaged with a 60 x oil immersion objective of the microscope and 2 x zoom magnification (120 x) controlled by FV10i-SW software. A 405 nm laser was used for excitation of DAPI and emission range from 420 to 520 nm was collected under the same exposure conditions for each sample. The image size was 1024 x 1024 pixels and the images were analyzed by FV10i -ASW ver.4.2c software. Thickness of one layer was 1 μm, and 10 Z-slices which include the bottom to top of neurons were used for merged image.

### Thrashing rate

The thrashing rate of adult *C*. *elegans* exposed to 254- or 222-UVC was examined by counting the number of body-bend (right-left is counted as a single occurrence) per minute scored in liquid (in 1× phosphate-buffered saline) under microscope [24]. The experiment was conducted on 10–28 worms for each treatment group. The mean number of thrashes ± standard error (SE) for each group was calculated and the observations were evaluated statistically and represented graphically. One-way NOVA was performed to test statistical significance.

### Neurodegeneration assays

Neuronal networks of synchronously cultured NC1686 (2-day-old (D2) adult) or TG2435 (D3 adult) were imaged by confocal microscope (FLUOVIEW FV10i, Olympus) 10 min and 4 h after UVC irradiation. Worms were imaged with a 60 x oil immersion objective of the microscope controlled by FV10i-SW software. A 473 nm laser was used for excitation of GFP and emission range from 490 to 590 nm was collected. The image size was 1024 x 1024 pixels and the images were analyzed by FV10i -ASW ver.4.2c software. Thickness of one layer was 1 μm, and Z-slices which include the bottom to top of neurons were used for merged image. For PVD neuron analysis, we observed zone 1 (anterior) and zone 2 (posterior) of body as described in E’s paper [25]. The number of puncta in PVD and CEP dendrites was counted visually from the captured z-stack images. One-way ANOVA was performed to test statistical significance.

### Harsh touch response

A total of 15–25 WT N2 (Day two adult) were analyzed 4 h after UVC irradiation. The head of the forward-moving worm was touched with a platinum wire, and the number of backward body bends was counted with a stereomicroscope (OLYMPUS SZ61) as described in Giles’s paper [26]. One-way ANOVA was performed to test statistical significance.

### Statistical analysis

All data were expressed as mean ± Standard Error (SE). One-way ANOVA was used to determine whether there are any statistically significant differences among the mean of control and treated samples.

## Results

### CPDs are induced not only by 254-UVC but also by 222-UVC

**Fig 1:**
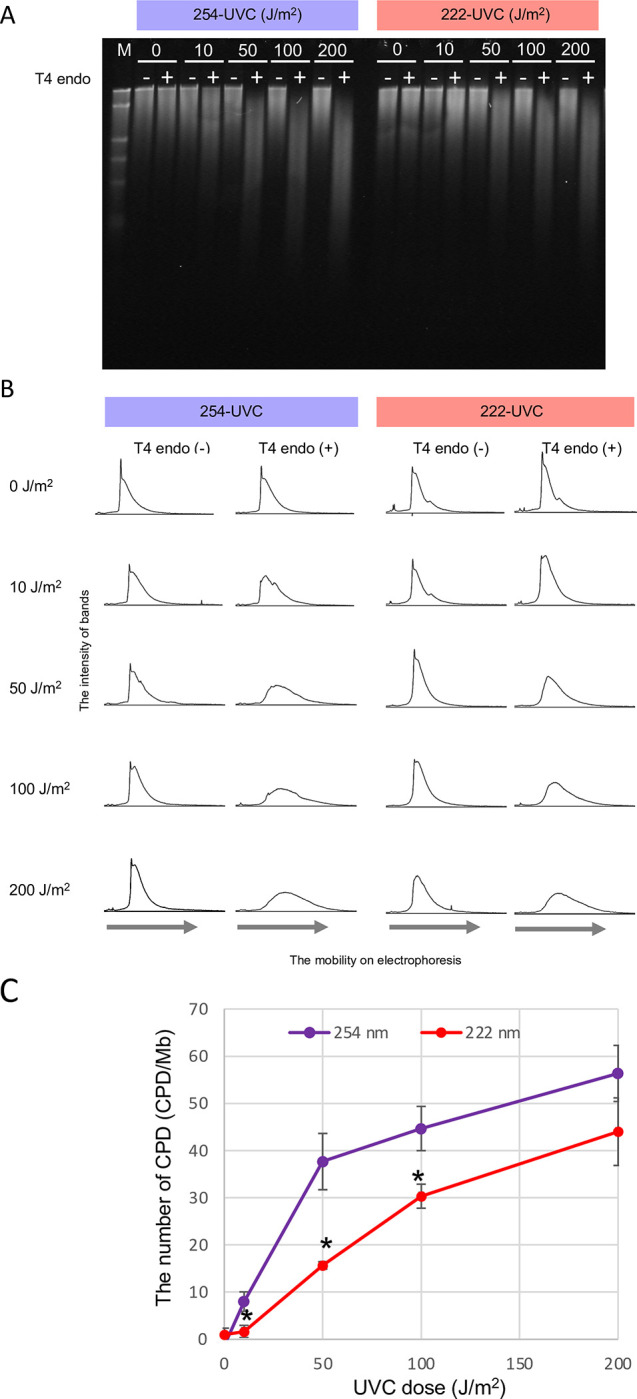
Frequency of CPDs induced by UVC irradiation on lambda DNA. (A) Detection of CPD levels on lambda DNA. Lambda DNA was irradiated by 254- or 222-UVC and the DNA was incubated with (+) or without (-) T4 endonuclease. DNA was analyzed in an alkaline gel. M, size marker (lambda-*Hin*d III cut). (B) Representation of DNA bands in the form of peaks. (C) The frequency of CPDs is calculated by the frequency of CPDs untreated with T4 endonuclease has been subtracted from the frequency of CPD treated with the enzyme. The purple and red lines indicate DNA damage frequency induced by 254-UVC and by 222-UVC, respectively. Error bars indicate the SE of three independent experiments. *P < 0.05 versus 254-UVC (one-tailed Student’s test).

Spectra emitted from a germicidal lamp for 254-UVC and a KrCl excimer lamp for 222-UVC were first measured. Results showed that both lamps effectively emitted only the characteristic single wavelength of 254 and 222 nm, respectively (S1A and S1B Fig). It is unknown whether 222-UVC induces CPDs, so the effects of 222-UVC on purified lambda DNA in water droplets were compared to 254-UVC. The lambda DNA solution was irradiated (10, 50, 100, and 200 J/m^2^) by 222- and 254-UVC. The DNA was digested by T4 endonuclease, a CPD glycosylase, to measure the amount of CPDs. Without T4 endonuclease treatment, lambda DNA electrophoresis bands were unchanged by 254-UVC or 222-UVC irradiation within 200 J/m^2^ (Fig 1A and 1B). Each sample was then treated with T4 endonuclease, revealing that 222-UVC irradiation induced 20%-50% less CPD damage than 254-UVC exposure. (Fig 1A–1C). These results indicated that, like 254-UVC, 222-UVC mainly induced CPD damage to DNA *in vitro*, but its activity was slightly reduced. A previous study has shown that unlike 254-UVC, 222-UVC induces little DNA photodamage in mouse skin or a three-dimensional (3D) skin tissue model [5]. This difference may be due to the limited penetration of 222-UVC into biological samples, unlike naked DNA in aqueous solution *in vitro*.

### 254- and 222-UVC differently affect the hatching rate

**Fig 2:**
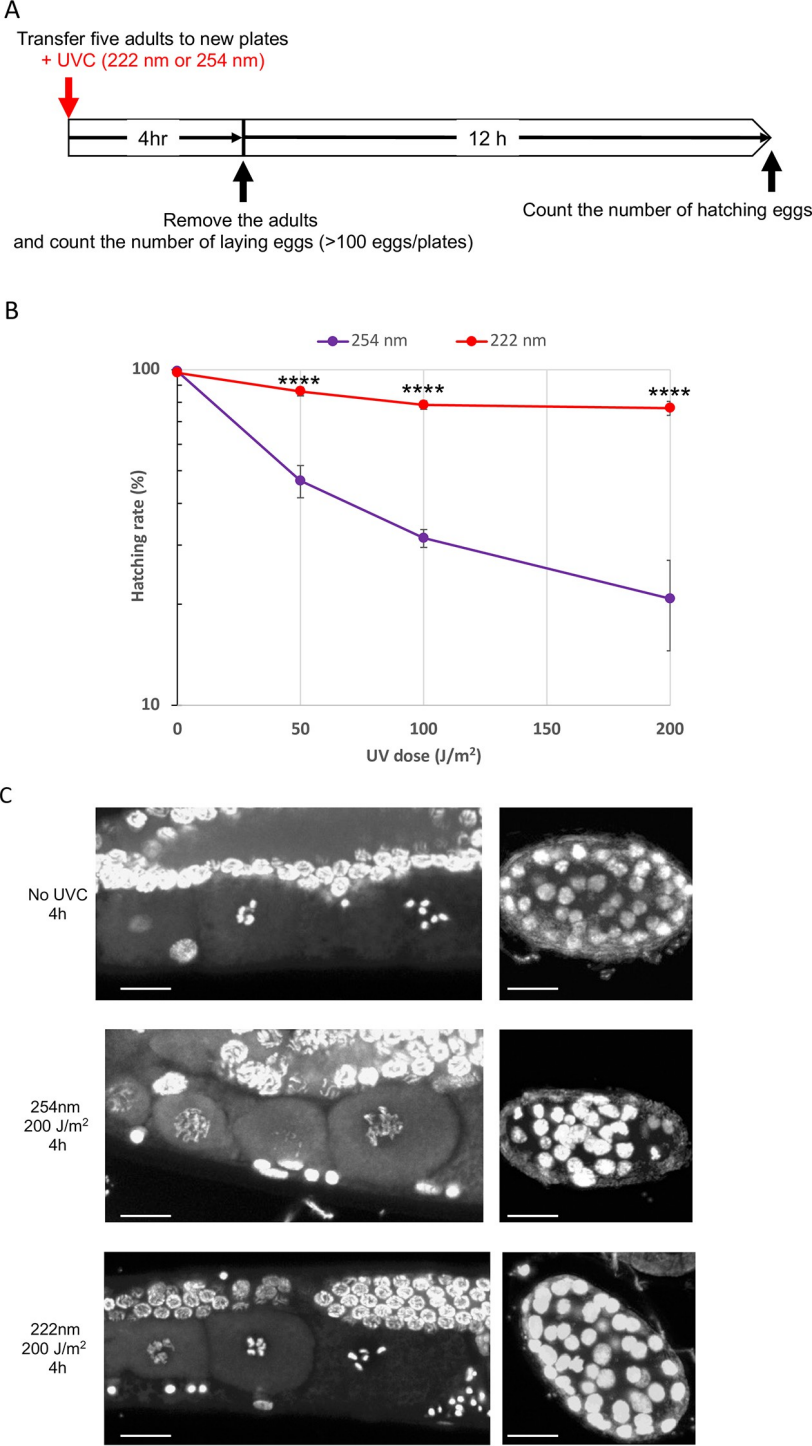
Effects of 254- and 222-UVC exposure on *C*. *elegans* hatching rate. (A) Schematic cartoon of experimental design. Five young gravid hermaphrodites (WT) were transferred to new NGM plates seeded with *Escherichia coli* and were irradiated with 50–200 J/m^2^ of 254- or 222-UVC. 4 h later, removed the adults and counted the number of laying eggs. 12 h later, counted the number of not-hatched eggs, and the hatching rate was calculated (hatching rate = the number of hatching eggs / the number of laid eggs x 100) (*n* = 60–200). (B) Survival of the eggs laid by WT hermaphrodites after 254- or 222-UVC. The purple line and red line indicate 254-UVC and 222-UVC survival, respectively. Error bars indicate the SE of six independent experiments. ****P < 0.0001 versus 254-UVC (two-tailed Student’s test). (C) The chromosome structure in full-grown oocytes (left panel) at diakinesis stage and the nuclei of early fertilized eggs (right panel) in the uterus 4 h later after UVC irradiation, was examined with DAPI staining. Bar indicates 10 μm.

The successive stages of oogenesis and early embryogenesis can be observed in a single, young gravid hermaphrodite (N2 WT day two adult). Each animal has ~15 fertilized eggs in the uterus, ~15 oocytes at diakinesis of meiosis I, ~10 oocytes at the diplotene stage, and >200 pachytene nuclei in the two gonads [23, 27]. To examine whether 254- and 222-UVC differently affect N2 fertilized eggs in the uterus, survival (hatching rate) of eggs laid 0–4 h after UVC irradiation was measured (Fig 2A). The hatching rate of eggs by 254-UVC decreased severely in a dose-dependent manner (Fig 2B). In contrast, the hatchability after 222-UVC did not decrease to ≤80%, even at 200 J/m^2^. Furthermore, 4 h after irradiation, the nuclei in early embryo in the uterus were visualized with DAPI staining. Results showed heterogeneous nuclear size and uneven brightness in eggs irradiated with 254-UVC, but almost uniform in 222-UVC and controls (Fig 2C right panel). These results showed that 254-UVC causes serious damage to the early embryo in the body, whereas 222-UVC is less severe. Interestingly, the chromosomes of fully grown oocytes appeared normal and bivalent chromosomes were clearly observed in control and 222-UVC irradiated animals, whereas in animals irradiated with 254-UVC 200 J/m^2^, aberrant and uncondensed chromosomes were observed (Fig 2C left panel). These results suggest that 222-UVC induced less DNA damage in oocytes and early embryos compared to 254-UVC irradiation in the *C*. *elegans* uterus.

### 222-UVC inhibits the motility of *C*. *elegans*

**Fig 3:**
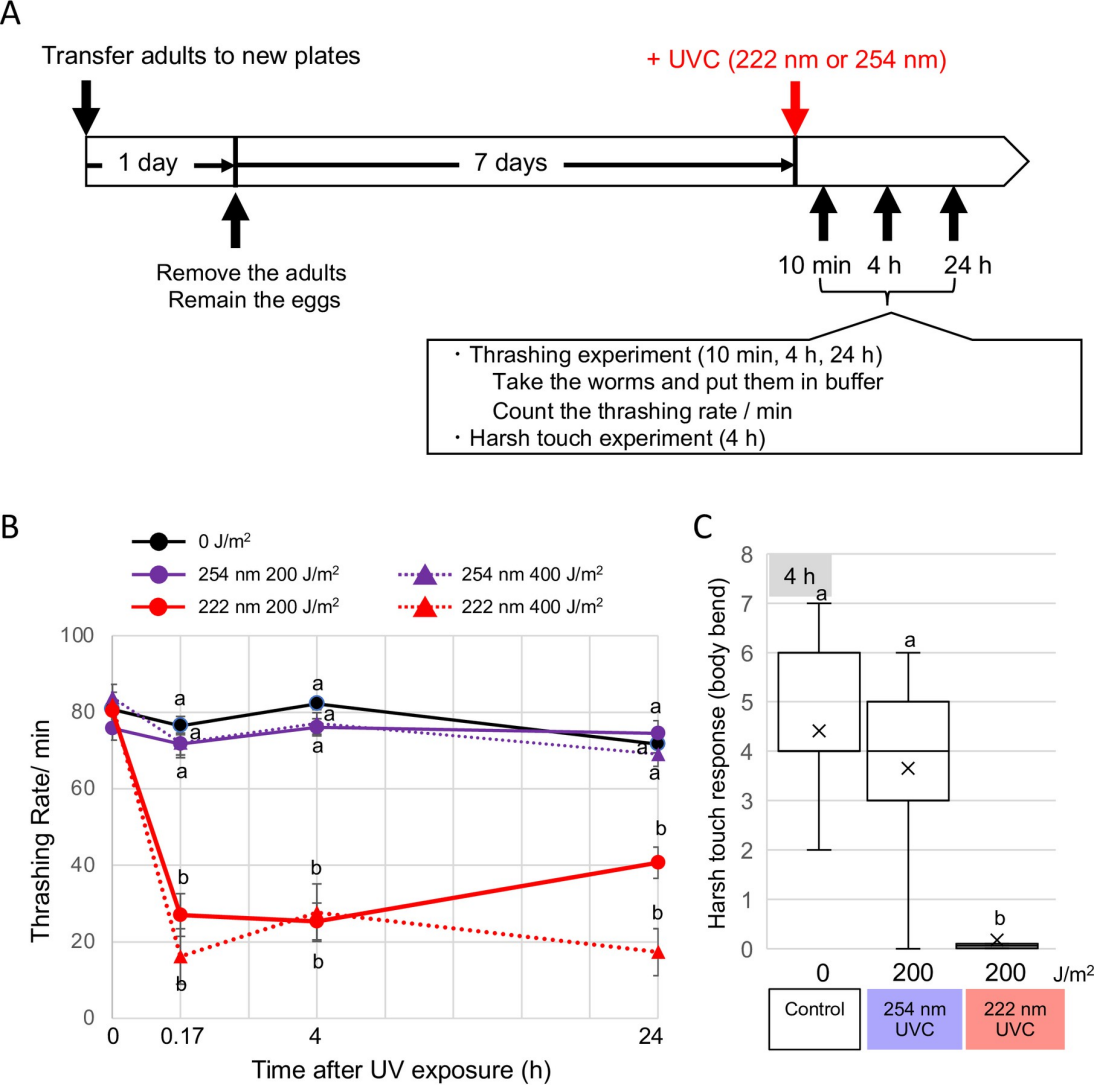
Effects of 254- or 222-UVC exposure on *C*. *elegans* motility. (A) Schematic cartoon of experimental design. Transferred five adults to new NGM plates seeded with *Escherichia coli*. One day later, removed the adults and remained eggs. 7 days later, took the adult worms and put them into PBS buffer and counted the thrashing number per minute. (B) The number of thrashes in 1 min was counted for each worm (*n* = 10–28 worms) at 0 min, 10 min, 4 h, and 24 h after UVC exposure. The purple line and red line indicate the thrashing rate of 254-UVC and 222-UVC irradiated worms, respectively. Colored solid and dotted lines indicate 200 and 400 J/m^2^ irradiation, respectively. Error bars indicate the SE for 10–28 worms. a and b indicate a significant difference between indicating two groups (one-way ANOVA followed by Dunnett’s method) (*P* < 0.05). (C) Harsh touch responses of WT animals. After 4 h from UVC exposure, response was assayed as the number of reverse body-bends a worm makes following a harsh touch stimulus to the head. Box plots show minimum, 25th percentile, median, 75th percentile, and maximum. Inside line and x indicate the value of median and average, respectively. a and b indicate a significant difference between indicating two groups (one-way ANOVA followed by Dunnett’s method) (*P* < 0.05).

In 222-UVC, where the hatching rate of eggs did not decrease so much, the motility of the parent worms was significantly impaired on a nematode growth medium (NGM) agar plate. To further investigate the motility of WT (Day two adult), changes in motility up to 24 h after either UVC irradiation were measured by the frequency of thrashing per minute in liquid (Fig 3A). After 24 h, the thrashing rate decreased slightly with age, even with the nonirradiated control, and there was no or little significant difference in the decrease with 254-UVC up to 400 J/m^2^ (Fig 3B). In contrast, 222-UVC dramatically reduced their motility. With 200 J/m^2^ or 400 J/m^2^ irradiation, the thrashing rate decreased to less than one-fourth at 10 min after irradiation. That is, 222-UVC mainly impairs the motility of *C*. *elegans* but not the survival of eggs in the body and vice versa in 254-UVC.

Next, we observed harsh touch response on agar plates to assess the effect of neuronal damage. The number of backward body bends following a harsh touch to the head of the worms was measured. In control worms, harsh touch resulted in normal reverse movement (4–6 body bends) (Fig 3C). After 4 h of 200 J/m^2^ 254-UVC exposure, the reverse locomotion after harsh touch was slightly decreased. In contrast, the reverse movement of worms irradiated with 200 J/m^2^ 222-UVC was significantly reduced. The neuronal damage induced by 222-UVC irradiation appears to cause motor inhibition.

### 222-UVC-induced neurodegeneration of sensory neurons in *C*. *elegans*

**Fig 4:**
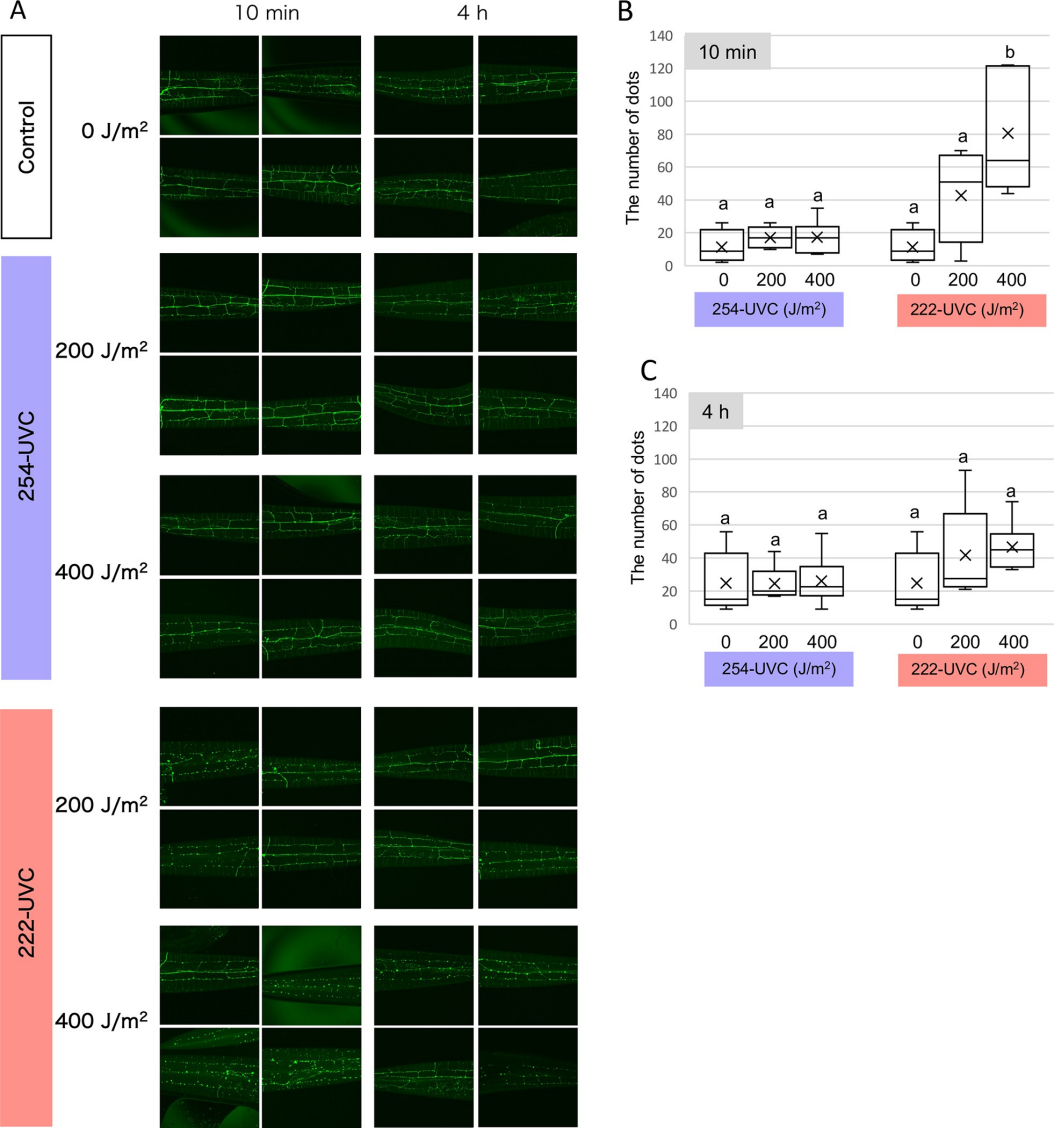
Sensory neurons degenerate upon UVC exposure. (A) Representation of damage in sensory neurons after UVC exposure. 3D reconstruction of confocal fluorescence from sensory neurons in a PVD::GFP (F49H12.4::GFP) transgenic line (NC1686). GFP-labeled sensory neurons were observed at 10 min and 4h after 254- and 222-UVC exposure (200 and 400 J/m^2^). (B and C) The number of GFP dots along sensory neurons. GFP dots were counted by looking with adding the mark on the pictures opened by ImageJ software. Box plots show minimum, 25th percentile, median, 75th percentile, and maximum. Inside line and x indicate the value of median and average, respectively. a and b indicate a significant difference between indicating two groups (one-way ANOVA followed by Dunnett’s method) (*P* < 0.05).

Because 222-UVC irradiation decreased not only thrashing activity but also a response to harsh touch stimuli, we next observed PVD sensory neurons involved in harsh touch response and exhibiting extensive branching along the body wall from neck to tail [28–30]. Using NC1686, which is visualized by the bright GFP signal in PVD neurons [20] the number of puncta (bead/bubble-like structures) along the dendrites of the PVD was counted to determine the level of neurodegeneration. After 10 min of 222-UVC exposure, the number of puncta and fragmentation increased dose-dependently (Fig 4A and 4B). After 4 hours of exposure to 222-UVC, punctual abnormalities remained high, but the difference was no longer significant (Fig 4A and 4C). In contrast, 254-UVC showed few puncta or fragmentation under 200 and 400 J/m^2^ conditions, almost the same as the nonirradiated control group (Fig 4A–4C).

**Fig 5:**
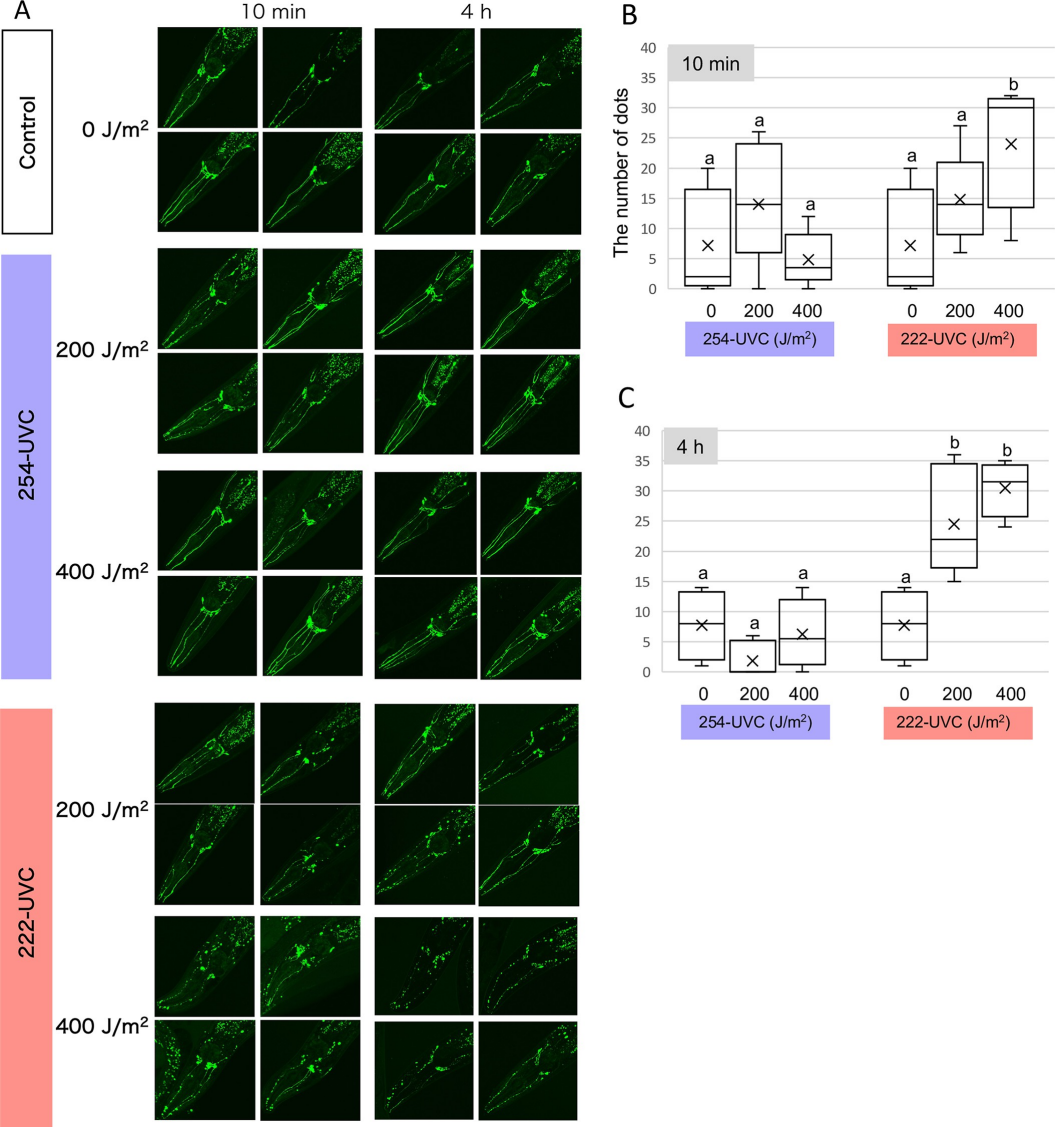
Dopaminergic neurons degenerate upon UVC exposure. (A) Representation of damage in dopaminergic neurons after UVC exposure. 3D reconstruction of confocal fluorescence from head dopamine neurons in a p*dat-1*::GFP transgenic line (TG2435). GFP-labeled dopaminergic neurons were observed 10 min and 4 h after 254-and 222-UVC exposure (200 and 400 J/m^2^). (B and C) The number of GFP dots along CEP dendrites in the head zone. GFP dots were counted by looking with adding the mark on the pictures opened by ImageJ software. Box plots show minimum, 25th percentile, median, 75th percentile, and maximum. Inside line and x indicate the value of median and average, respectively. a and b indicate a significant difference between indicating two groups (one-way ANOVA followed by Dunnett’s method) (*P* < 0.05).

To examine the effect of 222-UVC on other sensory neurons, we observed the dendrites of CEP and ADE dopaminergic neurons using the TG2435 strain, which expresses GFP under the control of the dopamine-transporter gene *dat-1* [21]. In the case of 254-UVC, 10 min or 4 h after 200 J/m^2^ exposure, CEP and ADE neurons were seen like in the no-irradiation control (Fig 5A–5C). Increasing irradiation dose did not change the structure and signal intensity of CEP and ADE neurons. In contrast, more puncture structures along CEP and ADE dendrites were observed at 10 min and 4 h after 200 J/m^2^ irradiation in the case of 222-UVC (Fig 5A). The number of puncta in CEP dendrites was shown in Fig 5B and 5C. With an increased dose of 222-UVC (400 J/m^2^), these defects were exacerbated, leading to the shortening or disappearance of neuronal extensions.

All image analyses of these neural networks were observed under the same exposure conditions for both 10 min and 4 h at different UVC wavelengths, so there was little effect of GFP photobleaching by 222- and 254-UVC exposures. Together, these results suggested that 254-UVC induces little neurodegeneration in PVD and CEP, whereas 222-UVC induces severe damage to both neurons located near the body surface. Similar bead/bubble-like structures have been generally observed in aging or injured *C*. *elegans*, and these structures were used as a marker of neurodegeneration in both invertebrate and vertebrate models [31–33].

### Simulation analysis of 222- and 254-UVC absorption depending on the depth of the biomaterial

The 254-UVC resulted in a reduced hatching rate of eggs but did not affect sensory neurons. In contrast, although 222-UVC did not affect the hatching rate, it inhibited the motility of worms and induced neuron degeneration. How are such differences observed between 254- and 222-UVC? The differences can be explained in terms of the distinct difference in the absorption coefficients for 222- and 254-UVC.

When UV light passes through a sample, its intensity exponentially decays, as in the following Eq (1):

$$I(x)=I_{0}\;exp(-\alpha x)(Lambert‐Beer\;law),$$ (1)

where *I*(*x*) is the penetrated light intensity at *x* μm depth from the surface, *I*_0_ is the light intensity at the surface (0 μm depth from the surface), *α* is the absorption coefficient (μm^−1^), and *x* is the distance from the surface (μm).

The linear absorption coefficient (*α*) represents the degree of the exponential decay of the intensity. A large coefficient value represents that UV light becomes significantly absorbed as it passes through a given medium, whereas a small value represents that UV light is hardly absorbed. To get the *α* values (in biomaterial) for 254- and 222-UVC, absorbance data of Lembares et al. [34] was used. They used porcine corneas as a biomaterial. The *α* values were calculated using Equation (2B) in Lembares’s paper [34]. The *α* for 222-UVC (0.225–0.269 μm^−1^) was 10 times higher than that of 254-UVC (0.0219 μm^−1^), meaning that 222-UVC is easily absorbed in the porcine corneas [34]. Next, it was thought that the difference between the effects of 254- and 222-UVC on *C*. *elegans* is dependent on the *α* values and the tissue depth.

**Fig 6:**
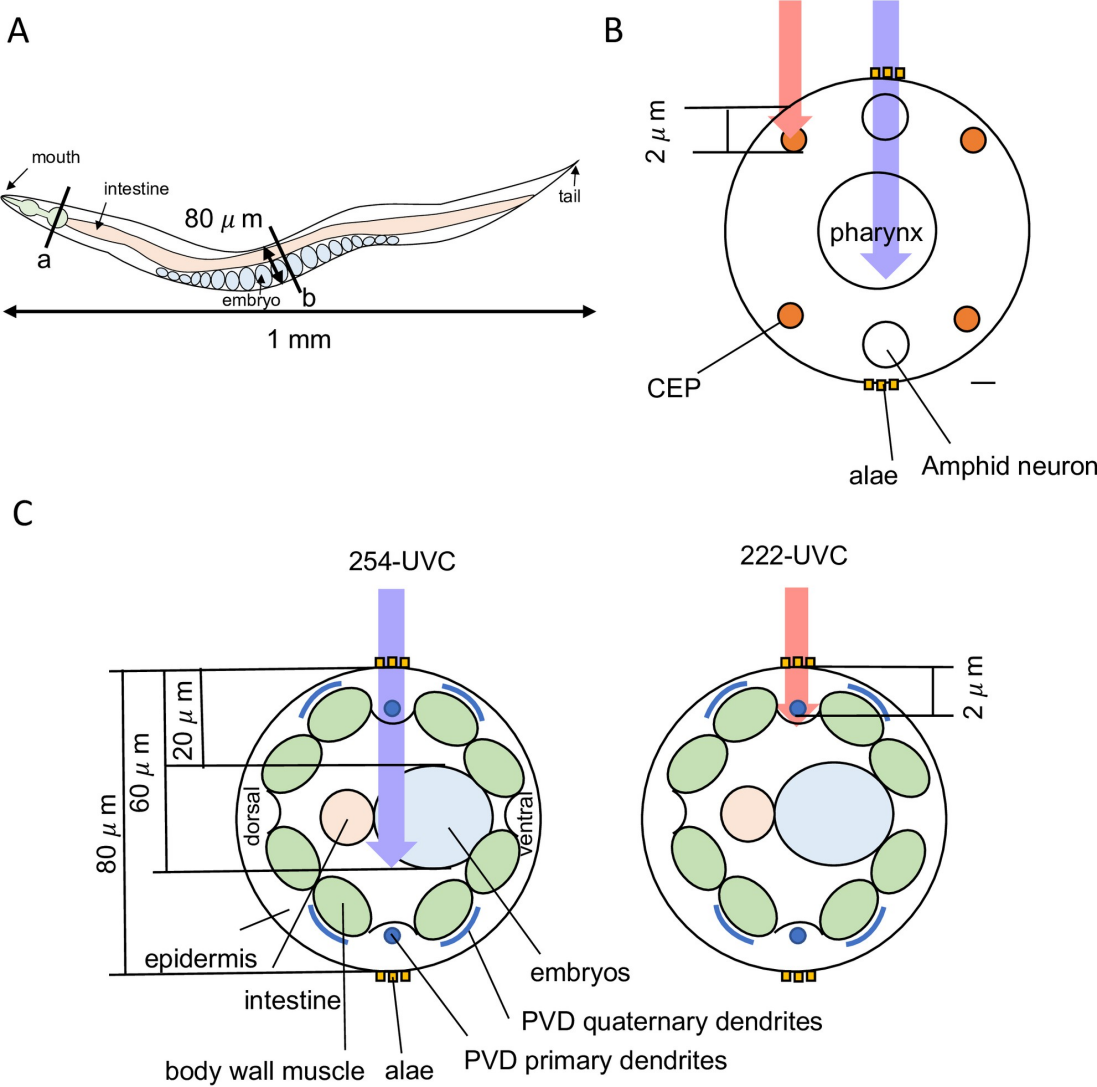
*C*. *elegans* structure. (A) *C*. *elegans* body plane, showing the side view. (B) Head section of *C*. *elegans* (cross section of “a” in Fig 6A). Orange circles show CEP neurons. Scale bar, 1.0 μm. (C) Cross-section of the midbody region (cross section of “b” in Fig A). Blue circles and lines show PVD neurons. Purple and pink arrows show 254- and 222-UVC, respectively. Images modified from those found at www.wormatlas.org. Altun, Z.F., L.A. Herndon, C. Crocker, R. Lints, and D.H. Hall (eds), 2002–2015 WormAtlas. Available at: http://www.wormatlas.org.

**Fig 7:**
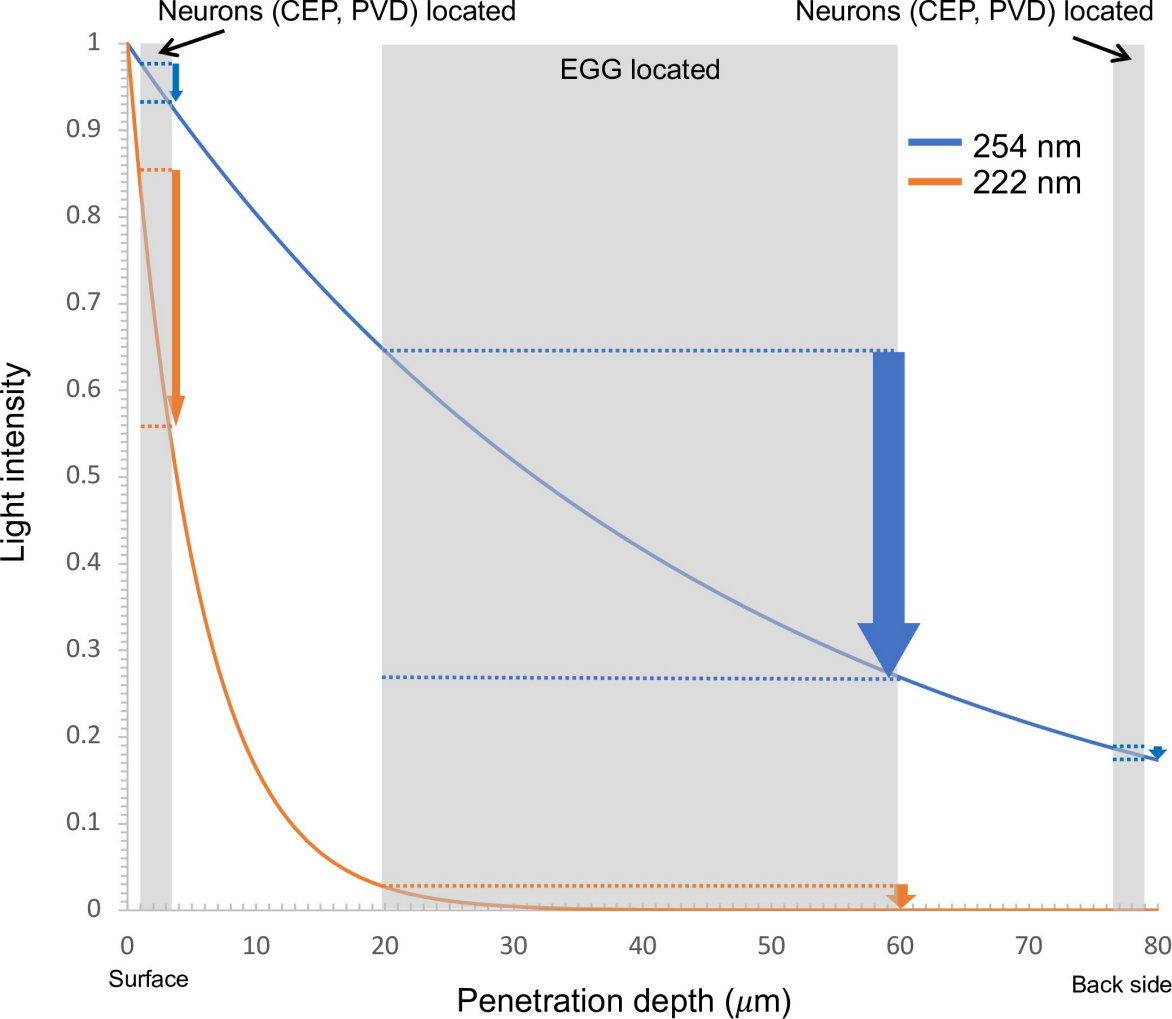
Relationship between light intensity and penetration depth for 254- and 222-UVC. The light intensity exponentially decays when it passes through a biomaterial. Blue line show the light intensity of 254-UVC, and orange line show the light intensity of 222-UVC, calculated with the absorption coefficient from the data [34]. Dot lines indicate where the light reaches the beginning and end of the tissue. Blue and orange arrows indicate the UVC energy absorbed in the relevant region for 254-UVC and 222-UVC, respectively. Gray areas show where neurons (CEP, PVD), and eggs are located.

The nematode’s body length is ~1 mm, and its body diameter is 50–80 μm (Fig 6A). The CEP neurons of *C*. *elegans* are positioned in the head and very close to the surface (~2 μm deep from the surface; Fig 6B) [35, 36]. The PVD neurons have highly ordered dendritic branches, and these dendrites grow at the interface of muscle and epidermal cells; therefore, PVD neurons are located at the very shallow surface of their body (1–2 μm deep from the surface; Fig 6C) [37]. When *C*. *elegans* crawls on agar plates, it uses alae, which runs along the left and right sides of the body and promotes locomotion on the plates, meaning that *C*. *elegans* was irradiated by UVC light from the side (Fig 6C). Therefore, eggs are located 20–60 μm deep from the surface (Fig 6C). Fig 7 describes the intensity decay of UV light passing through *C*. *elegans* as a function of penetration depth using Eq (1) and the *α* values for 254- and 222-UVC obtained earlier. In Fig 7, at a depth of 1–2 μm (the position of CEP or PVD neurons), the light intensity of 222-UVC is dramatically decreased, showing that a large amount of 222-UVC energy was transferred to this region (indicated by orange and gray arrows at 1–2 μm depth in Fig 7). Thus, CEP and PVD neurons can be severely damaged by 222-UVC. In contrast, those neurons are hardly damaged by 254-UVC (indicated by a blue arrow at 1–2 μm depth in Fig 7) because the 254-UVC intensity is not so decreased at a depth of 1–2 μm. In contrast, at a depth of 20–60 μm (the position of eggs), the light intensity of 222-UVC is almost zero (indicated by orange and gray arrows at 20–60 μm depth in Fig 7); therefore, eggs do not get damaged. At the position, 254-UVC is largely decreased (indicated by a blue arrow at 20–60 μm depth in Fig 7), meaning that eggs get more damaged by 254-UVC.

**Fig 8:**
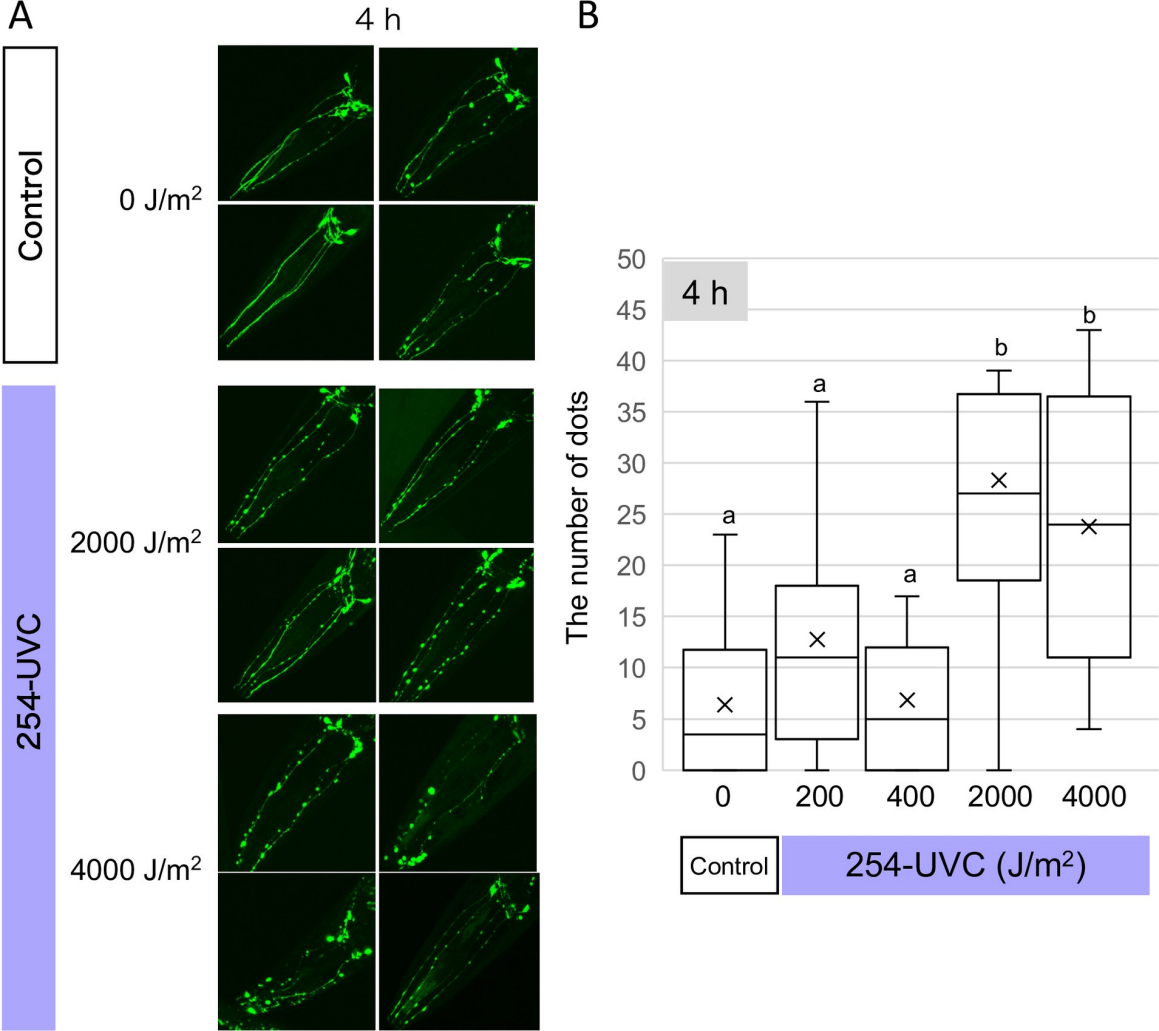
Dopaminergic neurons degenerate upon strong 254-UVC exposure. (A) Representation of damage in dopaminergic neurons after UVC exposure. 3D reconstruction of confocal fluorescence from head dopamine neurons in a p*dat-1*::GFP transgenic line (TG2435). GFP-labeled dopaminergic neurons were observed 4 h after 254-UVC exposure (2000 and 4000J/m^2^). (B) The number of GFP dots along CEP dendrites in the head zone. GFP dots were counted by looking with adding the mark on the pictures opened by ImageJ software. Box plots show minimum, 25th percentile, median, 75th percentile, and maximum. a and b indicate a significant difference between indicating two groups (one-way ANOVA followed by Dunnett’s method) (*P* < 0.05).

If this idea is correct, neurodegeneration may be observed when worms are exposed to high doses of 254-UVC. To test this hypothesis, TG2435 *pdat-1*::GFP was irradiated with 2000 or 4000 J/m^2^ of 254-UVC. As expected, a similar increase in the number of puncta was observed 4 h after 2000 and 4000 J/m^2^ 254-UVC irradiation as with 200 or 400 J/m^2^ 222-UVC irradiation (Fig 8A and 8B). These observations indicate that neurons on the body surface do not absorb as much energy as 222-UVC unless the dose of 254-UVC is increased. This result suggests that the distinct effects of 254-UVC and 222-UVC are due to their different absorption efficiencies on cells and tissues.

## Discussion

Far-UVC is a very attractive technology because it does not just reduce the transmission risk of a variety of microbial pathogens, including drug-resistant bacteria and inactivating viruses, and it is minimally hazardous for the human skin and eyes [38]. Many reports have shown that far-UVC does not easily induce UV-associated DNA lesions in rats, mice, and human keratinocytes in a 3D human skin model [5–7, 38, 39]. So, people are trying to use a far-UVC lamp for killing SARS-CoV-2 and other viruses instead of a 254-UVC lamp [39, 40]. However, this study using lambda DNA *in vitro* showed that far-UVC (222-UVC) also induced CPD formation as did 254-UVC. Therefore, the effects of 222-UVC on viruses and microorganisms, including COVID-19, are believed to act primarily as DNA-damaging agents. Several papers have been published on the effects of 254-UVC irradiation on nematodes [23, 41, 42]. In addition, the amount of CPD produced *in vivo* by nematode 254-UVC irradiation has also been investigated [43]. Therefore, in the future, CPD damage caused by 222-UVC in *C*. *elegans* may also be detected in the cell nucleus near the body surface by observing it using the *in situ* method.

The *C*. *elegans* adult hermaphrodites have 302 neurons that belong to an independent nervous system of the large somatic nervous system (282 neurons) and the small pharyngeal nervous system (20 neurons). In the somatic nervous system, neurons and their networks are located between the hypodermis and the body wall muscles and the basement membrane with the hypodermis [44]. Even with such a small number of nervous systems, *C*. *elegans* can perform complex behaviors, such as chemotaxis, thermosensation, and associative learning, in addition to basic locomotion, foraging, and defecation [45]. It is likely that the neurodegeneration of C. elegans indued by 222-UVC irradiation was the cause of the inhibition of motility.

We also reported recently that 222-UVC causes severe damage to guard cells and epidermal cells of *Arabidopsis thaliana* [19]. These results suggested that 222-UVC has capabilities that negatively affect the surface of plants or small animals, such as *C*. *elegans*. Our simulation analysis showed that the 222-UVC energy is almost absorbed in a shallower depth, but the 254-UVC energy is absorbed in a deeper depth. The simulated results were consistent with our experimental results that 222-UVC affects *C*. *elegans* surface neurons and 254-UVC affects the DNA of oocytes and early embryos localized in the body. These conclusions further support previous reports that far-UVC light (200–225 nm), unlike conventional UV germicidal light (254 nm) efficiently kills both drug- resistant and drug-sensitive microbes without the mammal skin damaging effects [5, 38, 39, 46]. Our studies also show that 222-UVC can be used for various biomedical applications that would require the reduction of surgical site infection, without the need of additional protective equipment. Furthermore, it is known that UV irradiation leads developing melanoma [47], but our research may show the possibility that 222-UVC irradiation can be used for the treatment of melanoma. Far-UVC light has the potential uses of variety of biomedical applications.

How does 222-UVC affect sensory neurons in the nematode? The inhibition of motility of worms by irradiation of 222-UVC is so quick (10 min after exposure). Fig 1 clearly shows that 222-UVC exposure can induce CPDs, which prevent DNA transcription and translation. Blocked translation biologically affects many processes; however, it takes some time. Therefore, it is difficult to say that CPD formation on genomic DNA is the reason for the quick inhibition of motility. The UV irradiation induces reactive oxygen species (ROS), including superoxide radicals, hydrogen peroxide, and hydroxyl radical [48, 49]. Due to unpaired electrons, ROS are highly reactive and oxidize multiple cell targets, including oxidative damage to proteins, DNA, and lipids resulting in the disruption of cellular functions [50]. There is much literature reporting a significant connection between ROS and neurodegenerative diseases (Parkinson’s disease, Alzheimer’s disease, Huntington’s disease, etc.) [51, 52]. Oxidative damage and aging can increase the neurodegenerations of dendrites of CEP and PVD neurons [25, 53, 54]. Taken together, ROS induced by 222-UVC may cause neurodegeneration and neuronal damages observed in sensory neurons in *C*. *elegans*.

Radiotherapy with high-energy charged particles has become an attractive therapy for cancer cells because this approach causes less damage to nearby healthy tissue than conventional photon therapy [55]. The exquisite dose localization of charged particles allows higher radiation doses to be given to tumor tissue, whereas normal tissues are exposed to lower doses. Such a concept has not been thought in UV field so far. As radiotherapy for human, we can use the difference in the properties of both UVCs (222- and 254-UVC) for plants or small animals to damage specific organs. For instance, when damaging tissues in a deeper depth (embryo in nematodes, etc.), 254-UVC is suitable for irradiation; when damaging tissues in a shallow depth (sensory neurons of nematodes, epidermal cells of plants, etc.), 222-UVC is suitable. Characterizing the effects of 222-UVC on living organisms requires a thorough understanding of its wavelength properties, which opens up the possibilities of using 222-UVC.

## Supporting information

S1 Fig Spectra of the light sources used in this study. (A) Spectra of a germicidal lamp (254-UVC; Toshiba Electric) at a distance of 20 cm. (B) Spectra of a 15 W KrCl excimer lamp (222-UVC) at a distance of 12.5 cm. 254- and 222-UVC sources emitted radiation at 6.8 and 1.7 J/m^2^ s, respectively.
(TIF)

S1 Raw image (PDF)
