# Supplementary figures and images for: 222 nm far-UVC efficiently introduces nerve damage in Caenorhabditis elegans
Source: PLoS One. 2023 Jan 31;18(1):e0281162. doi: 10.1371/journal.pone.0281162 (PMC9888708; doi:10.1371/journal.pone.0281162)

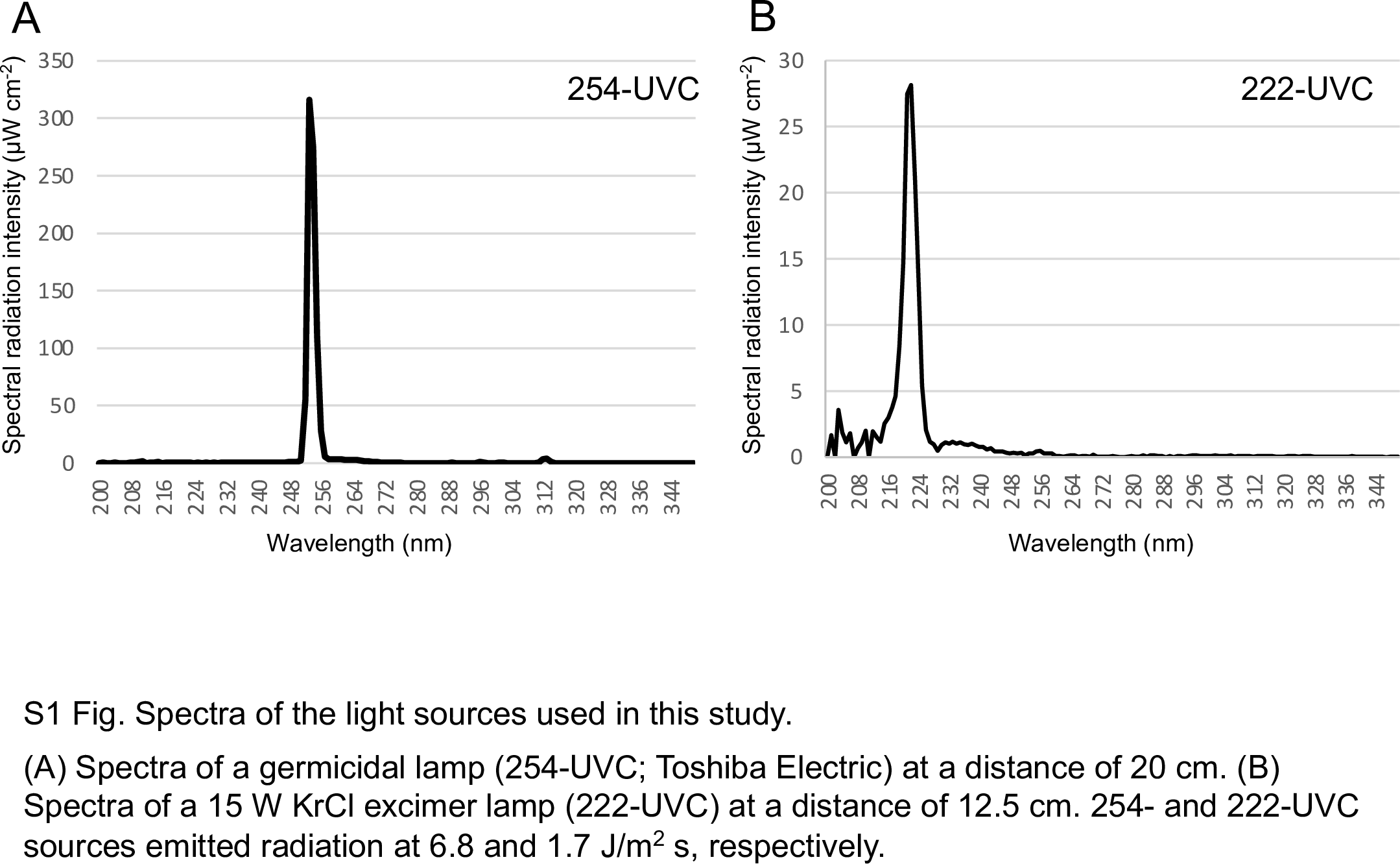

Supplement: S1 Fig — (A) Spectra of a germicidal lamp (254-UVC; Toshiba Electric) at a distance of 20 cm. (B) Spectra of a 15 W KrCl excimer lamp (222-UVC) at a distance of 12.5 cm. 254- and 222-UVC sources emitted radiation at 6.8 and 1.7 J/m2 s, respectively. (TIF) [file pone.0281162.s001.tif]
