# Supplementary material for: 222 nm far-UVC efficiently introduces nerve damage in Caenorhabditis elegans
Source: PLoS One. 2023 Jan 31;18(1):e0281162. doi: 10.1371/journal.pone.0281162 (PMC9888708; doi:10.1371/journal.pone.0281162)

M: Size Marker (lambda-Hind III)
1: 254 nm 0 J/m2 - T4 endo
2: 254 nm 0 J/m2 + T4 endo
3: 254 nm 10 J/m2 - T4 endo
4: 254 nm 10 J/m2 + T4 endo
5: 254 nm 50 J/m2 - T4 endo
6: 254 nm 50 J/m2 + T4 endo
7: 254 nm 100 J/m2 - T4 endo
8: 254 nm 100 J/m2 + T4 endo
9: 254 nm 200 J/m2 - T4 endo
10: 254 nm 200 J/m2 + T4 endo
11: 222 nm 0 J/m2 - T4 endo
12: 222 nm 0 J/m2 + T4 endo
13: 222 nm 10 J/m2 - T4 endo
14: 222 nm 10 J/m2 + T4 endo
15: 222 nm 50 J/m2 - T4 endo
16: 222 nm 50 J/m2 + T4 endo
17: 222 nm 100 J/m2 - T4 endo
18: 222 nm 100 J/m2 + T4 endo
19: 222 nm 200 J/m2 - T4 endo
20: 222 nm 200 J/m2 + T4 endo

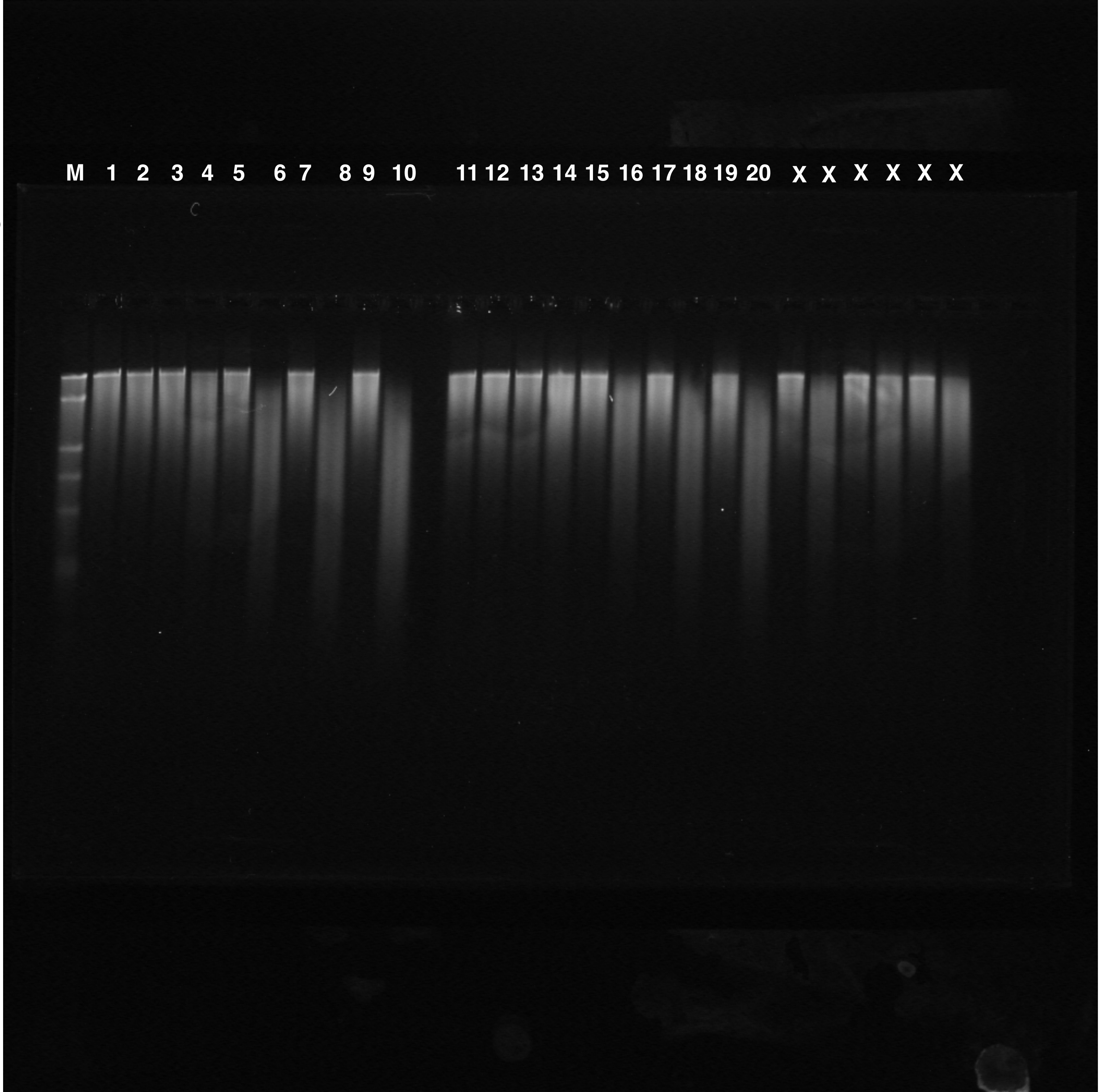

Supplement: S1 Raw image — (PDF) [file pone.0281162.s002.pdf]
